# Emodin: an alveolar macrophage protector in acute pancreatitis induced lung injury

**DOI:** 10.7150/ijms.105965

**Published:** 2025-03-31

**Authors:** Zhe Chen, Xuanchi Dong, Yongwei Song, Bowen Lan, Yalan Luo, Haiyun Wen, Hailong Chen

**Affiliations:** 1 Department of General Surgery, The First Affiliated Hospital of Dalian Medical University, Dalian, Liaoning, P.R. China.; 2 Department of General Surgery, Third People's Hospital of Dalian, Dalian Medical University, Dalian, Liaoning, P.R. China.; 3 Laboratory of Integrative Medicine, The First Affiliated Hospital of Dalian Medical University, Dalian, Liaoning, P.R. China.; 4 Institute (College) of Integrative Medicine, Dalian Medical University, Dalian, Liaoning, P.R. China.

**Keywords:** alveolar macrophage, mitochondrial damage, mitophagy dysfunction, mitochondrial membrane potential

## Abstract

**Background:** Emodin (EMO), an anthraquinone derivative from roots and leaves of various plants, has been widely used in many inflammatory diseases. Alveolar macrophages (AMs) play a critical role in maintaining alveolar homeostasis in the lung. However, the comprehensive mechanisms of EMO therapy on AMs during acute pancreatitis-associated lung injury (AP-ALI) have not been reported.

**Methods:** Both in vivo [caerulein/ lipopolysaccharides (LPS)-induced AP-ALI in mice] and in vitro MH-S models were generated to assess the protective features of EMO on mitochondrial damage and mitophagy dysfunction of AMs during AP-ALI progression.

**Results:** First, in vivo, the relative quantity of AMs was significantly decreased with time in AP-ALI mice; however, the mitochondrial flux presented earlier changes than the relative quantity of AMs in our experimental system. EMO pretreatment significantly alleviated the severity of lung injury and improved the damaged alveolar structure, reversing mitochondrial impairment in AMs. Secondly, in vitro, EMO significantly enhanced mitophagy and alleviated mitochondrial damage. Furthermore, the results following mitophagy inhibition by 3-methyladenine (3-MA) demonstrated that the protective effects of EMO were partially achieved by manipulating the mitophagy-mitochondria-alveolar macrophage axis.

**Conclusion:** These data enabled a more comprehensive understanding of the therapeutic effects of EMO in AP-ALI.

## Introduction

Acute pancreatitis (AP) is a frequent reason for emergency department visits 1. While mild-to-moderate forms of AP are associated with self-limiting systemic inflammatory responses in over 2/3 of cases, severe AP (SAP) continues to be a critical illness that requires protracted hospitalization, prolonged time in intensive care and a slow recovery process 2, 3. AP is associated with systemic complications, even persistent (multiple) organ failure. In fact, acinar cell death, infectious inflammation, intestinal mucosal barrier injury, as well as the inflammatory cascade could be the underlying pathophysiological mechanisms of AP 4. Acute lung injury (ALI)/acute respiratory distress syndrome (ARDS) is the most involved complication 5. Furthermore, uncontrolled inflammation of the lungs is commonly regarded as the primary etiology of ALI/ARDS 6.

Autophagy is a complex process of intracellular degradation of senescent or malfunctioning organelles. The selective autophagy of mitochondria, known as mitophagy, is critical for eliminating damaged mitochondria 7 and preserving the integrity of the mitochondrial network 8. Due to the predominantly oxygen-dependent energy production of mitochondria, their capacity to generate adenosine triphosphate (ATP) is significantly affected when mitophagy dysregulation occurs, resulting in cellular dysfunction 9. Studies have shown that mitochondrial dysfunction in macrophages is involved in diseases, such as myocardial infarction, vascular injury, and colitis disease 10-12. Meanwhile, dysfunctional mitochondria trigger severe inflammatory responses, which exacerbate lung injury and multiple organ failure 13. Alveolar macrophages (AMs), a predominant immune cell localized in alveoli in humans and rodents, serve as sentinels in the lung alveolar during homeostasis. AMs can either amplify inflammation or isolate contamination 14. We therefore hypothesized that mitochondrial dysfunction or damage in AMs could be one of the predominant factors during the pathophysiological progression of acute pancreatitis-associated lung injury (AP-ALI).

Emodin (EMO) is a natural product that belongs to the anthraquinone derivative family. Recent studies have suggested that EMO exhibits an extensive range of pharmacological characteristics, encompassing anti-cancer, hepatoprotective, anti-inflammatory, antioxidant and antimicrobial effects 15, 16. It has been demonstrated that EMO exhibits anti-inflammatory properties 17, specifically by regulating NLR family pyrin domain containing 3 (NLRP3) inflammasome activation 18. However, whether EMO impacts mitophagy or regulates the correlation of mitochondrial dysfunction apart from NLRP3 inflammasome activation during AP-ALI remains poorly understood.

In the present study, a caerulein/LPS-induced AP-ALI mouse model was used to investigate how EMO influences alveolar macrophage distribution and mitochondrial damage in AMs. Meanwhile, in vitro macrophage models (MH-S macrophages) were used to explore the protective effects of EMO on the correlation between mitophagy dysfunction and mitochondrial damage. Furthermore, a mitophagy inhibitor was used to confirm whether EMO's protective effects potentially function by enhancing mitophagy activation. The purpose of the present study is to obtain a comprehensive understanding of EMO's therapeutic effects on maintaining AM homeostasis in AP-ALI.

## Materials and methods

### Materials

Information on antibodies, experimental models, chemicals, peptides, recombinant proteins, critical commercial assays, oligonucleotides and software are presented in Tables SI-III.

### Animals

C57BL/6 male mice (12 weeks) were fed with a standard chow diet and housed in a temperature- and humidity-controlled facility. The animal experiments were conducted and authorized by the Research and Animal Ethics Committee of Dalian Medical University. The experiments were carried out in accordance with the ARRIVE guidelines.

### AP-ALI mouse model

As depicted in Figure 2A, the acute pancreatitis model and workflow diagram illustrate that the mice were segregated into four groups. Acute pancreatitis was induced by intraperitoneal injection of caerulein (100 μg/kg, i.p. per hour x10), which has been extensively documented 19, and 10 mg/kg LPS (E. coli serotype O55:B5) was intraperitoneally injected right after the last injection of caerulein. Control mice only received vehicles (5 ml/kg, 1x PBS). EMO (40 mg/kg body weight 20) was administered twice (right before the first dose of caerulein, and 2 h after the last dose of caerulein) through gastric gavage. Mice were anesthetized with 3% isoflurane mixed with air through a small animal respiratory anesthesia machine at 2 and 24 h post-injection with LPS. Once each mouse was confirmed to be deeply anesthetized, 200-300μl of mouse blood was collected from the posterior orbital venous plexus. As orbital blood collection was performed as a terminal procedure, the mice were euthanized by spinal dislocation. The entire pancreas tissue and the lower lobe of the right lung were used for pathological analysis. The right middle lobe was used for frozen section preparation of lung tissue, and the right anterior lobe was used for dry-wet ratio calculation. It is important to note that all mice underwent in vivo labeling with macrophages before being included in the experimental group. Considering the effects of performing BALF on tissue injury and AMs in the lung, it is necessary to perform parallel modeling of mice requiring BALF collection.

### Histopathological examination

Fresh pancreatic and lung tissue samples were fixed by immersion in formalin, then embedded in paraffin and sectioned to an approximate thickness of 4 µm. After dewaxing and dehydration procedures, the tissue sections were stained using hematoxylin-eosin (HE) staining. A light microscopic examination was then performed to assess the pathological changes in the pancreas and lung tissues. Based on previous literature 21, 22, mouse pancreas and lung histopathology were evaluated.

### AMs in-vivo labeling technique

PKH26 phagocytic cell labeling was performed, according to the manufacturer's instructions and previous reports 23, 24. Briefly, a 100-μM solution of PKH26 was diluted to a concentration of 0.5 μM using diluent B. Mice were subjected to anesthesia using 3% isoflurane for 3 min. Subsequently, the mice were promptly hung by their incisors at a 60˚ angle and 75 μl of a 0.5 μM PKH26 solution was accurately transferred into the posterior part of the throat using a pipette. The movement and behavior of animals was then observed inside the cages until they walked normally.

### Cell culture

MH-S cells (MH-S is a macrophage, alveolar cell line that was isolated from the lung of a 7-week-old, male mouse) were maintained in RPMI-1640 medium supplemented with 10% FBS, and were cultured at 37˚C in a humidified 5% CO_2_ incubator.

### Isolation of AMs in murine bronchoalveolar lavage fluid (BALF)

Murine AMs were harvested from the BALF of C57BL/6 mice at 2 and 24 h post-injection with LPS. Mice were anesthetized with 3% isoflurane mixed with air through a small animal respiratory anesthesia machine. Once it was confirmed that the mice were deeply anesthetized, they were euthanized by spinal dislocation. The trachea was exposed and cannulated with a 24G catheter (Wego). Ice-cold PBS (0.8 ml) with 100 μM ethylenediaminetetraacetic acid (EDTA) was gently infused, followed by extraction. This was repeated 5 times. A total volume of 3.5-3.8 ml/mouse was collected. Next, BALF fluid was centrifuged at 1,500 RPM at 4˚C for 10 min. The supernatants were collected and assessed by spectrophotometer for turbidity experiments. After being subjected to ACK lysis (BD Biosciences) for 2 min, the cell pellets were washed in 2% FBS and stored on ice until staining. Protein in supernatants was quantified using a Bicinchoninic acid assay (BCA) protein assay kit, and total cholesterol was quantified using a mouse cholesterol kit, according to the manufacturer's instructions.

### Flow cytometry

Cells isolated from BALF were stained with 200 nm MitoTracker™ Green FM for 20 min. Following incubation with Fixable Viability Stain 700 (BD Biosciences), cells were incubated using a blocking antibody (2.4G2; BD Biosciences) for 5 min, according to the manufacturer's instructions. Cells were then stained with specific antibody cocktails at 4˚C. Negative Control Compensation Particles Set was also performed. For MH-S cells, samples were assessed using the LSRFortessa cell analyzer (BD Biosciences) and FlowJo software.

### Cell viability assay

The cell viability of MH-S was assessed using cell counting kit-8 (CCK-8) assay. In summary, MH-S cells were seeded in 96-well plates at ~1x10^5^ cells/well. A total of 12 h later, cells were treated with indicated concentrations of EMO for 6/12 h, and incubated with 10 μl of CCK-8 solution at 37˚C for 30 min. At the indicated wavelength, the mixture's absorbance was measured.

### Transmission electron microscopy (TEM)

Cells were fixed at room temperature with 2.5% glutaraldehyde (Absin). They were then rinsed three times with PBS and subjected to an escalating progressive sequence of ethanol treatments for 8 min. Samples were then infiltrated with resin and polymerized. The images were acquired with TEM.

### ELISA

The levels of interleukin-1 beta (IL-1β) and tumor necrosis factor-alpha (TNF-α) in either serum, BALF or cultured supernatants were determined using an ELISA kit, according to the manufacturer's instructions.

### Western blotting

Cells were lysed with RIPA buffer containing a protease inhibitor cocktail (NCM Biotech). Cell lysate was centrifuged at 12000 g for 15 min at 4˚C. The supernatant fluid is the total cell lysate. Protein concentrations were measured using a BCA protein assay kit (NCM Biotech). Equivalent amounts of protein from each sample were diluted with loading buffer, boiled for 6 min, subjected to SDS/PAGE (12% gels) at 100 mV and subsequently transferred to PVDF membranes. Membranes were blocked in 5% non-fat dried skimmed milk powder in TBST [Tris-buffered saline containing 0.1% (v/v) Tween 20] for 1 h at room temperature and were incubated overnight at 4˚C in 5% BSA, in TBST with anti-mouse antibodies at recommended concentration in the instructions. Following incubation with the appropriate horseradish peroxidase-conjugated antibodies for 1 h at room temperature, protein signals were observed using chemiluminescence detection, and the intensity was analyzed by ImageJ software (National Institutes of Health). Briefly, the ratios of integrated densities of the proteins (i.e. LC3II and LC3I) to GAPDH were calculated. Relative fold changes of LC3II and LC3I were presented by normalizing the ratio of each treatment group to the control group.

### Superoxide and mitochondrial membrane potential evaluation

Cells were treated with 3-methyladenine (3-MA; 5 mM for 1 h) and stimulated as previously described. MitoSOX red, a fluorescent indicator to specifically detect superoxide, producing red fluorescence and JC-1 fluorescent probe, an ideal fluorescent probe widely used to detect mitochondrial membrane potential, were assembled with each kit's instructions, respectively. Samples were then analyzed using flow cytometry as described above.

### DNA and RNA isolation, reverse transcription, and quantitative PCR (qPCR)

Mitochondrial D-loop-specific primer and nuclear DNA encoding Tert primer (Table S3) allowed us to determine the mitochondrial DNA (mtDNA)/nuclear DNA (nDNA) ratio. ONE-4-ALL Genomic DNA Mini-Preps Kit was used to extract and purify MH-S cellular DNA, according to the manufacturer's instructions. Total RNA was isolated using TRIzol reagent, and reverse-transcribed DNA was amplified using Monscript RTIII All-in-One Mix with dsDNase. RT-qPCR was performed with RapidStart Universal SYBR Green qPCR Mix using a LineGene 9600 Plus fluorescence quantitative thermal cycler (Hangzhou Bioer Co., Ltd.). Briefly, 1 μg total RNA was reverse transcribed with 5× RTIII All-in-One Mix at 37°C for 2 min, followed by 55°C for 15 min and 85°C for 5 min in a 20 μl reaction volume. Subsequently, each PCR reaction from the cDNA template (2 μl RT product or purified MH-S cellular DNA) was performed using specific primers (Table S3) at 95°C for 1 min, then 40 cycles at 95°C for 1 min, 60°C for 1 min. The relative expression level of the samples within each group was calculated using the 2- ΔΔ CT method, with β-actin or Tert as the internal reference.

### Immunofluorescent staining

Cells were fixed with 4% paraformaldehyde at 4˚C for 10 min and permeabilized using PBS (0.1% Triton-100). Following blocking with PBST (PBS plus 0.1% Tween-20) at 4˚C for 30 min, incubation with specific primary antibodies was performed at 4˚C overnight. Next, samples were incubated with appropriate second fluorescent antibodies (Alexa-488, Alexa-568) and DAPI. The MitoTracker Green FM (200 nM) and LysoTracker Red (50 nM) were used, according to the manufacturer's instructions. All images were obtained using an Olympus BX63 confocal microscope system (Olympus Corporation).

### Wet/dry weight (W/D) ratio calculation

Briefly, the upper part from the right side of the lung was removed and weighed immediately for wet weight measurement. Next, tissue was placed in an oven at 70˚C for 24 h to completely dry the moisture. The dry weight of the lung tissue was evaluated, and the W/D ratio was obtained.

### Statistical analysis

The PRISM 9.0 was used to perform statistical analysis. Unless specified otherwise in the image caption, all results from in vitro and in vivo experiments are presented as the mean ± standard deviation. An unpaired Student's t-test was used to analyze differences between the two groups, and a one-way ANOVA was conducted for groups with multiple variables Tukey's method was used for pairwise comparisons when ANOVA was used. P<0.05 was considered to indicate a statistically significant difference.

## Results

### EMO mitigates caerulein/LPS-induced AP-ALI in mice

**Figure 1:**
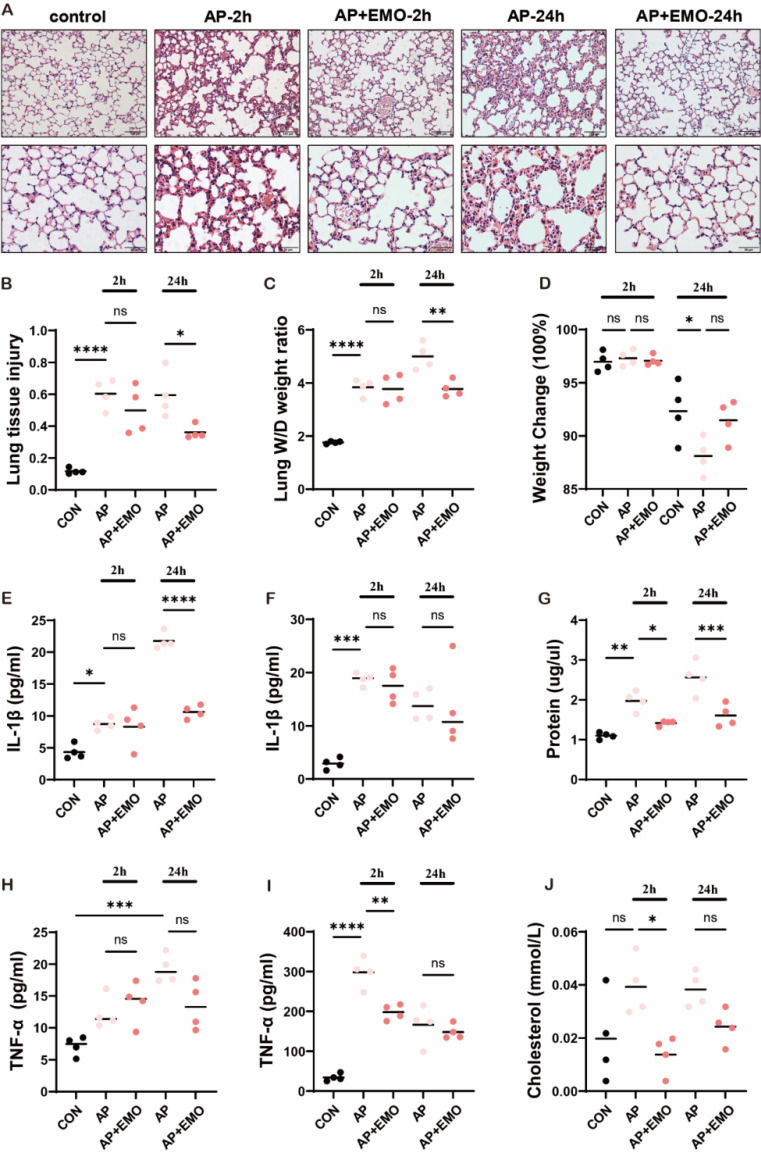
EMO mitigated caerulein/LPS-induced AP-ALI in mice. (**A**) Representative images of H&E staining in the lung with or without EMO treatment in AP-ALI mice (n=4 per group). (**B**) Quantification of lung tissue injury (n=4 per group). (**C**) Analysis of lung W/D weight ratio (n=4 per group). (**D**) Measurement of body weight with or without EMO treatment (n=4 per group). Expression levels of IL-1β in (**E**) BALF and (**F**) serum were evaluated by ELISA (n=4 per group). (**G**) Protein level assessment in BALF (n=4 per group). Expression levels of TNF-α in (**H**) BALF and (**I**) serum were evaluated by ELISA (n=4 per group). (**J**) Measurement of cholesterol level in BALF (n=4 per group). *P<0.05, **P<0.01, ***P<0.001 and ****P<0.0001.

To investigate how EMO protects and impedes the progression of AP-ALI, a mouse model of AP-ALI was first generated using a combined injection of caerulein plus LPS. The successful induction of AP was confirmed through histopathological assessment (Figure S1A) and examination of lipase levels (Figure S1B and C) at different time points (Figure S1A-C). The histopathological staining results (Figure 1A and B) showed that EMO significantly decreased tissue damage in the lung: More intact alveolar structure, clear interspace, less inflammatory cell infiltration and thinner interalveolar spectrum, both at 2 and 24 h after the event. Furthermore, EMO had significant effects on the wet/dry ratio of lung tissue (an index of vascular permeability that indicates the severity of pancreatitis; Figure 1C and D). In addition, the data in Figure 1E-J suggested that EMO also dramatically decreased IL-1β and TNF-α secretion in the serum and BALF together with the altered cholesterol levels.

### EMO reverses the reduction of mitochondrial impairment in AMs in AP-ALI mice

**Figure 2:**
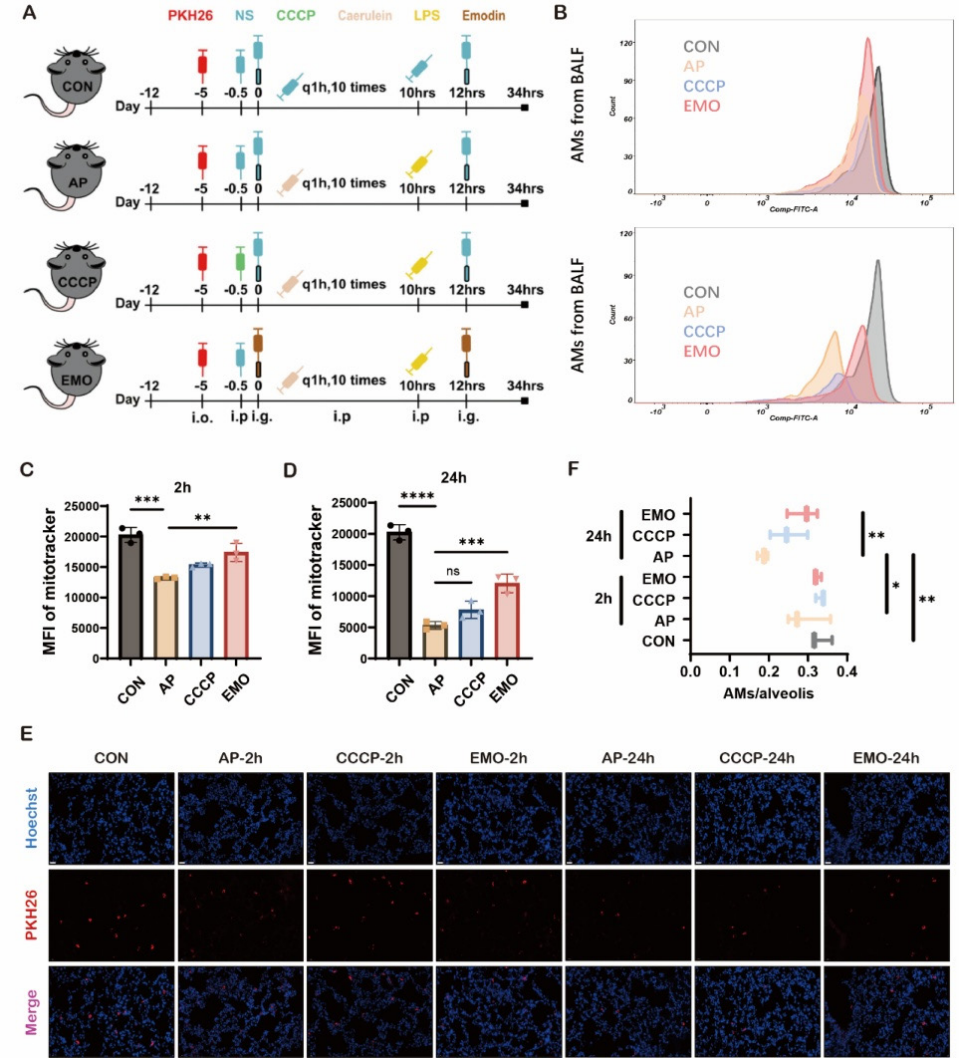
EMO impacts mitochondrial changes in AMs during acute pancreatitis. (**A**) Schematic of the acute pancreatitis model and workflow. (**B**) Flow cytometry for the assessment of mitochondrial density in BALF in control or paralleled experiments at 2 and 24 h after the model was established. (**C**) Quantification of MitoTracker fluorescence in AMs 2 h after caerulein and LPS injection. (**D**) Quantification of MitoTracker fluorescence in AMs at 24 h after caerulein and LPS injection. (**E**) Representative images of frozen sections in the lung from each group at the indicated time points. Hoechst (blue) marks the nucleus and red marks PKH26-AMs. Empty (black) spaces are alveolar spaces. Scale bars, 20 μm. (**F**) Quantification of AMs/alveolis from each group. *P<0.05, **P<0.01, ***P<0.001 and ****P<0.0001.

Next, the potential impact of EMO on native AMs during the AP-ALI we explored (Figure 2A). Of note, it was demonstrated that EMO or Carbonyl Cyanide m-Chlorophenyl Hydrazone (CCCP, a mitochondrial depolarizing chemical used as a positive control) treatment does not reverse the establishment of the mouse AP-ALI model (Figure S1A-C). It has been well-documented that AMs are abundant in the alveolar compartment of naive mice, residing exclusively on the alveolar side of the lung 24. The frequency of other immune cells was below 2%. First, AMs were isolated from BALF and subjected to a 10-min incubation with MitoTracker Green FM. Flow cytometry was subsequently conducted (Figure S1D). The data showed a reduction in mitochondrial density of AMs at 2 and 24 h after the event, compared to the control group. Of note, EMO administration reversed the changes of acute pancreatitis-associated mitochondrial density (Figure 2B-D). Next, AMs were labeled in vivo using PKH26PCL fluorescent cell linker system, due to their phagocytic capabilities. In the present experimental model, in BALF, over 97% of PKH26-labeled cells were AMs with a 90% efficiency rate. As ~60% of alveolar spaces were devoid of AMs in naive mice 17, the levels of AM/alveolar were measured among the four groups. The data showed that there is no significant difference at 2 h after LPS injection with or without EMO treatment. However, 24 h after LPS injection, fewer PKH26+ cells were observed in the pancreatitis group, compared with the control. Of note, the decrease in PKH26+ cells in mice with pancreatitis was reversed by CCCP and EMO (Figure 2E and F).

### EMO inhibits mitochondrial membrane potential in macrophages in vitro

**Figure 3:**
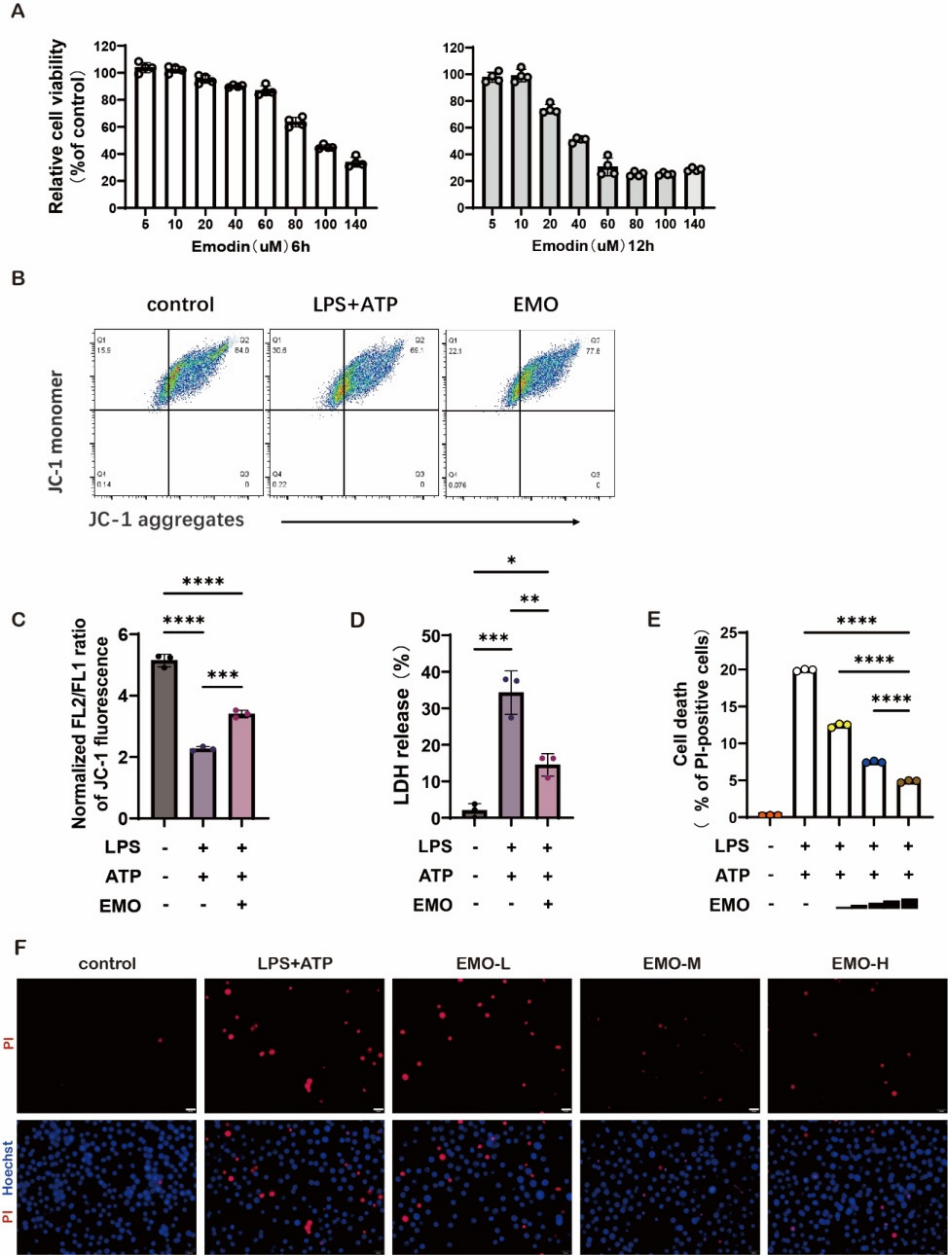
EMO changes the voltage of mitochondria and alleviates cell damage in MH-S. (**A**) MH-S cells were treated with ΕΜΟ (5-140 μM, 6 or 12 h), and then the cytotoxicity of the compound was determined using a CCK-8 assay (n=4 per group). (**B**) Analysis of mitochondrial membrane potential levels in MH-S by JC-1 fluorescence. n=3/condition. (**C**) Quantification of mitochondrial membrane potential from B. (**D**) Quantification of LDH in MH-S cultural supernatant from B. (**E**) The cell death immunofluorescence photographs were produced by staining with Hoechst 33342 (5 μg/ml, blue) and PI (2 μg/ml, red; same treated as in A). Scale bars, 20 μm. (**F**) Quantification of PI-positive cells in e. n=3/condition. *P<0.05, **P<0.01, ***P<0.001 and ****P<0.0001.

The lung macrophage cell line MH-S was used to evaluate the cytotoxicity of EMO in vitro using the CCK-8 assay, during which MH-S cells were treated with varying concentrations of EMO for either 6 or 12 h. The data showed that, compared with the control group, EMO presented no influence on the viability of MH-S at a dose of 20 μM (Figure 3A). Emerging studies have reported that the abnormality of mitochondrial membrane potential is tightly associated with the severity of inflammation in many inflammatory diseases. To investigate how EMO regulates mitochondrial voltage in macrophages, the JC-1 probe was used to assess the changes of mitochondrial membrane potential following stimulation with LPS plus ATP. The results showed that LPS/ATP stimulation significantly decreased the mitochondrial membrane potential and reduced JC-1 aggregation in mitochondrial matrix (Figure 3B). The existence of green fluorescence monomers in Figure 3A indicated a mitochondrial membrane potential abnormality in the LPS/ATP group. However, with 2 h of EMO pretreatment, there is an increase in mitochondrial membrane potential evidenced as the gradual transition of fluorescence color to red (the alternative mitochondria-specific markers, MitoTracker Deep Red and MitoTracker Green, were used to stand for the differentiation of healthy mitochondria). The statistical analysis in Figure 3C further confirmed the above findings. A decreased level of lactate dehydrogenase in the culture supernatants (Figure 3D) indicated the cytoprotection of EMO. In addition, based on the dual-labeling with propidium iodide and Hoechst, EMO exerted significant protective effects on cell death in MH-S in the stimulation of LPS/ATP (Figure 3E-F).

### EMO enhances mitophagy which leads to an alleviation of mitochondrial damage in macrophages in vitro

**Figure 4:**
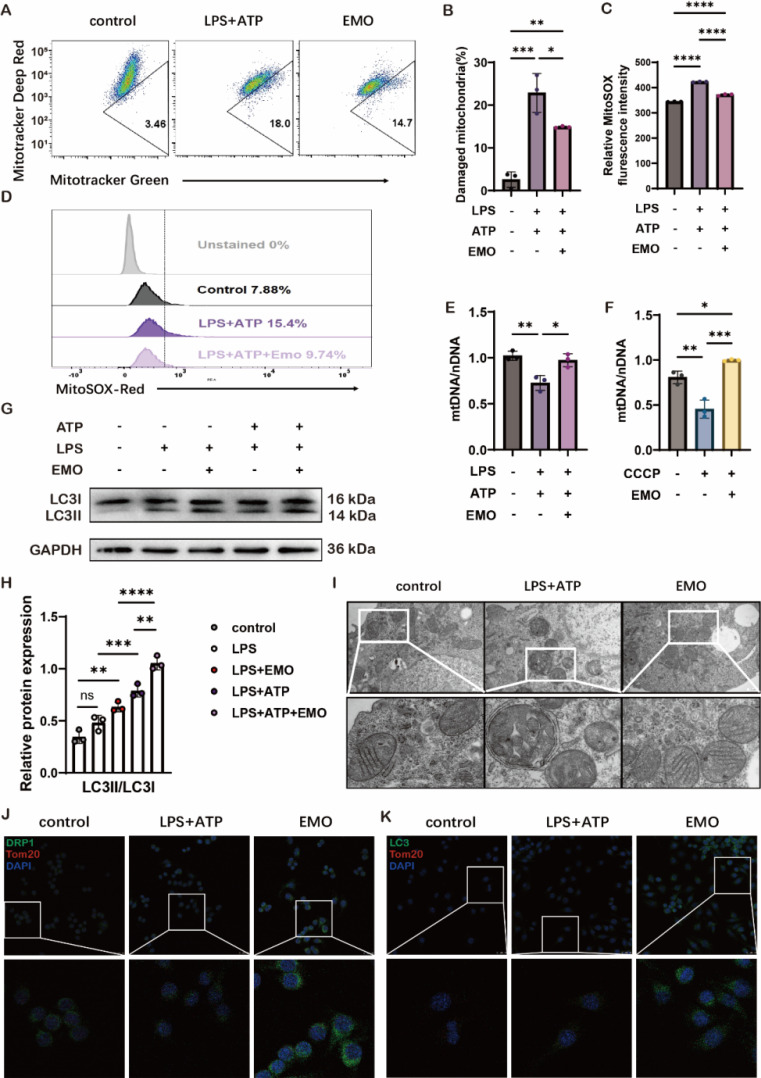
EMO induces mitophagy as a means to reduce mitochondrial injury. (**A**) MH-S mitochondrial membrane potential levels were analyzed using MitoTracker deep red and MitoTracker green staining. MH-S was pretreated with EMO (5μM, 2h), primed with LPS (500 ng/ml, 4 h), and followed by stimulation with ATP (2 mM, 1 h). n=3/condition. (**B**) Quantification of damaged mitochondria. (**C**) Quantification of MitoSOX. (**D**) Flow cytometry of MitoSOX (n=3 per condition). (**E**) The ratio of mtDNA to nDNA in MH-S was analyzed by qPCR to ascertain the mass of the mitochondria (n=3 per condition). (**F**) The ratio of nDNA to mtDNA in MH-S was treated for 2 h with or without EMO (5 μM) and CCCP (500 nM) (n=3 per condition). (**G**) Immunoblots of LC3I and LC3II on protein lysates from MH-S pretreated with/without various doses of EMO (5 μM, 2 h), primed with LPS (500 ng/ml, 4 h) and followed by stimulation with/without ATP (2 mM, 1 h; n=3 per condition). (**H**) Relative quantification of LC3II/LC3I from G. (**I**) The morphology of mitochondria in MH-S, both with and without EMO pretreatment and LPS/ATP treatment (n=3/condition). Scale bars, 0.5 μm. (**J**) Colocalization of DRP1 (green) and Tom20 (red) in MH-S (n=3 per condition). Scale bars, 25 μm. (**K**) Colocalization of LC3 (green) and Tom20 (red) in MH-S (n=3 per condition). Scale bars, 25 μm. *P<0.05, **P<0.01, ***P<0.001 and ****P<0.0001.

To explore EMO therapeutics on mitochondrial damage, dual labeling of MitoTracker Green positive and MitoTracker Deep Red negative staining were utilized, which represented the percentage of damaged mitochondria. The data in Figure 4A-B showed that LPS/ATP stimulation significantly increased damaged mitochondrial mass to 18%, as compared with 3.46% in the control group. However, 5 μM EMO pretreatment for 2 h markedly decreased damaged mitochondrial to 14.7% in MH-S cells. On the other hand, the combination of LPS/ATP stimulation induced an increase of reactive oxygen species (ROS) resulting from destroyed mitochondria (Figure 4C). EMO pretreatment could significantly reduce the ROS level based on the quantification and flow cytometry of MitoSOX in MH-S macrophages (Figure 4C-D). In addition, the ratio of mtDNA and nDNA was also assessed to investigate mitochondrial damage induced by LPS/ATP or CCCP stimulation, and the data in Figure 4E-F suggested a significant protective effect of EMO on increasing the mtDNA/nDNA ratio in MH-S macrophages, thereby avoiding mitochondrial damage. Mitophagy, a subtype of autophagy, plays a vital role in maintaining the balance of reactive oxygen species in mitochondria of macrophages. In the present study, an autophagy indicator, microtubule-associated protein 1A/1B-light chain 3 (LC3)II/LC3I as also assessed, and EMO pretreatment showed a marked effect on increasing LC3II/LC3I levels which indicated a protective effect of EMO in LPS/ATP-associated mitophagy disorder (Figure 4G-H). In addition, mitochondrial morphology (such as cristae, telltale signs of double-membrane autophagosome) was also improved following EMO pretreatment (Figure 4I). To further investigate the mitophagy alteration by EMO, another mitochondrial marker-TOMM20 was used in MH-S cells with LPS/ATP stimulation. In Figure 4J-K, the increase in colocalization of TOMM20 with both dynamin-related protein 1 (a key mediator of mitochondrial fission) and LC3 in MH-S cells indicated that EMO exhibited consistent protection over mitochondria. Collectively, the present data suggested that the protective effects of EMO in the LPS/ATP-stimulated in vitro macrophage model could enhance mitophagy, thus avoiding further mitochondrial damage.

### Selective inhibition of mitophagy reverses EMO's protective effects on mitochondrial damage in vitro

**Figure 5:**
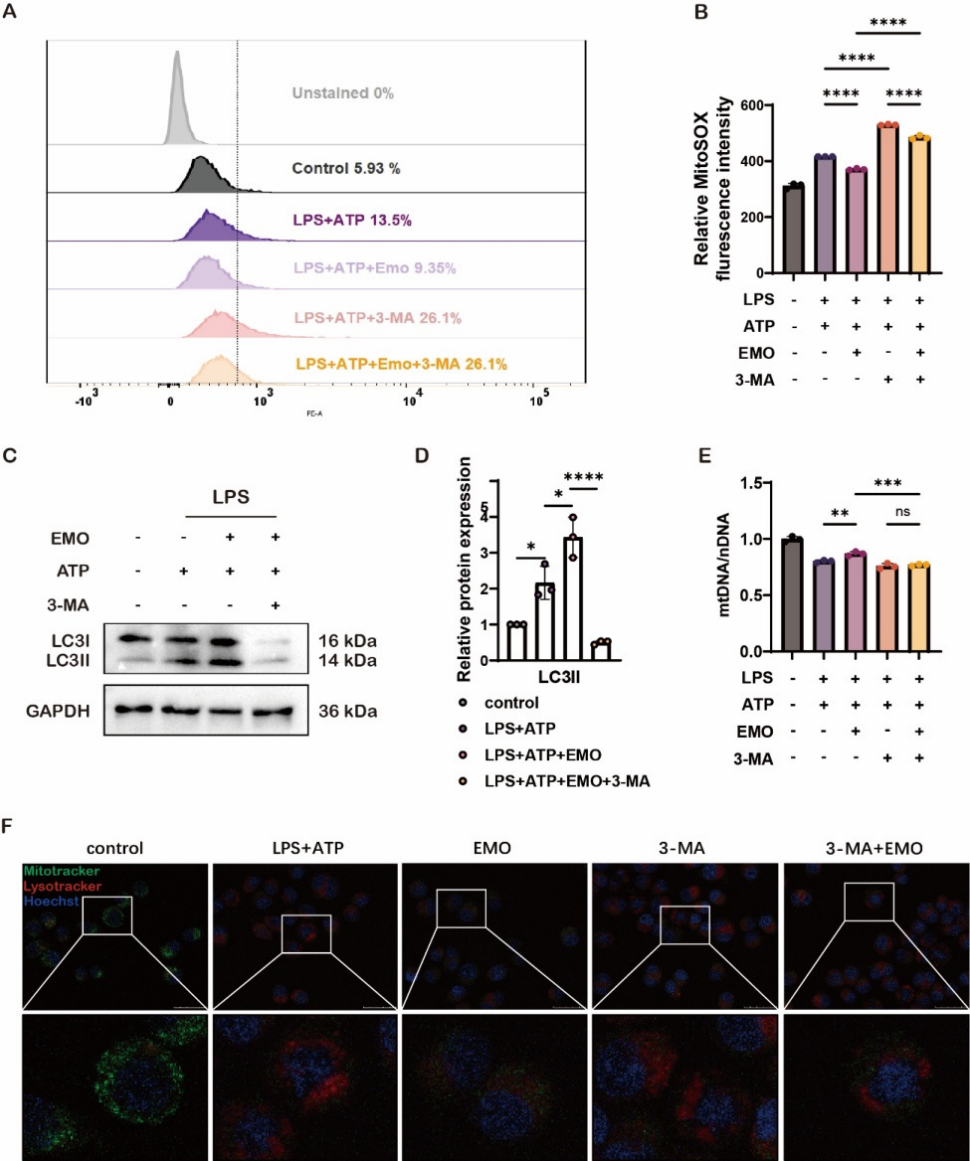
Inhibiting autophagy in murine macrophages counteracts EMO's protective effects against inflammation and mitochondrial damage. (**A**) Analysis of MitoSOX levels in MH-S cells were treated with 3-MA (5 mM, 1 h) before EMO (5 μM, 2h) treatment, LPS (500 ng/ml, 4 h) and ATP (2 mM, 0.5 h) treatment. (**B**) Quantification of relative MitoSOX fluorescence intensity. (**C**) Western blot analysis of LC3I/II protein expression in MH-S cells (n=3 per condition). (**D**) Relative quantification of LC3II from C. (**E**) The ratio of mtDNA to nDNA in MH-S was analyzed by qPCR to ascertain the mass of the mitochondria. (**F**) The colocalization of MitoTracker (green) and LysoTracker (red) in MH-S (n=3 per condition). Scale bars, 25 μm. *P<0.05, **P<0.01, ***P<0.001 and ****P<0.0001.

To elucidate whether EMO's protective effects involve regulating alveolar macrophage mitophagy during AP-ALI, an autophagy/mitophagy inhibitor, 3-methyladenine (3-MA), was used in vitro. Of note, 3-MA pre-treatment led to a significant increase in ROS and mtDNA levels, together with a high accumulation of damaged mitochondria following LPS/ATP stimulation, both in the 3-MA and EMO/3-MA groups in MH-S macrophages (Figure 5A, B and E). Specifically, 3-MA treatment significantly inhibited the EMO-induced increase in LC3II and LC3I expression in MH-S cells, which suggested that EMO-related protective effects in macrophages functioned by regulating the mitophagy pathway (Figure 5C-D). Notably, the observation of the colocalization of MitoTracker and LysoTracker indicated that EMO enhanced mitophagy activity and alleviated mitochondrial damage; however, 3-MA treatment led to a significant decrease in MitoTracker signals and an increase in LysoTracker amount in Figure 5F. These findings suggested that EMO's protective effects against mitochondrial damage in macrophages are potentially associated with the regulation of mitophagy dysfunction.

## Discussion

To date, the pathophysiological mechanism of systemic injury in AP remains poorly understood. It has been well documented that the lung is the most frequently involved organ associated with AP-induced systemic damage 25. Emerging studies showed that the profiles of monocytes and macrophages are differentially altered during the pathogenesis of pancreatitis 26, 27. AMs monitor the luminal surface of the epithelium, together with epithelial cells, contributing to setting the threshold and the quality of the innate immune response in the lung mucosa 28. On the other hand, the overactivation of AMs could also induce some tissue damage; it is therefore necessary to elucidate how AMs are involved in AP-ALI. With the unique phagocytic characteristic of macrophages, they were marked with PKH26 fluorescence in vivo before being observed in frozen slices. This technique allowed us to not only track the macrophage location but also measure the number of labeled macrophages in situ compared with the amount of macrophages in the alveolar space.

Previous studies found that AMs efficiently intercept particles, bacteria and foreign materials, contributing to alveolar defense and clearance. Nevertheless, under the condition of a decrease in the AM-to-alveoli ratio, the "self-cleaning" ability of AMs would markedly decrease in inflammatory diseases such as acute pancreatitis, etc. Specifically, in the initial stage of AP-ALI, once the alveolar equilibrium was disturbed, molecules from the collectin family (i.e. SP-A, SP-D and C1q) would facilitate phagocytosis and inflammation by binding the apoptotic cells via calreticulin-CD91 in AMs. Of note, there is a critical time range for the contact of alveolar epithelium with deceased cells, bacteria, fungi and particles. The process of those contacts will induce the release of multiple chemokines and the recruitment of neutrophils. In the present study, a single AM was detected in approximately every three alveoli in the AP model, which indicated a marked decrease with time, despite having less of an impact on the resident AMs in the early stage of AP. Based on these results, we hypothesized that the number of resident AMs could be selected as a potential marker for the severity of AP-ALI. Using flow cytometry, mitochondrial levels were assessed in resident AMs. The results showed that a decrease in the mitochondrial level was observed 2 h after caerulein-induced acute pancreatitis in the mouse model, and a continuous reduction was observed until 24 h after the event, which seemed to continue with the progression of lung injury. It has been demonstrated that mitochondrial play a critical role in regulating inflammatory responses through their genuine damage-associated molecular molecules (DAMPs), which serves as a physical scaffold for activating pattern recognition receptors 9. Specifically, mitochondria provide a distinctive framework for DAMP redistribution, PRR signaling and inflammation response to cellular stress; on the other hand, these processes can stimulate innate and adaptive immune reactions to maintain homeostasis 13. However, during the inflammatory cascading amplification of AP, a considerable number of surface receptors were markedly increased. Meanwhile, multi-cellular responses, such as autophagy and caspase 4/11, were also activated in response to RCD-associated mitochondrial outer membrane permeabilization to maintain homeostasis in AMs 29. Additionally, related studies confirm that AMs mitochondrial metabolic reprogramming under stress, alongside changes in their immune state 30. Further comparison of the present results indicated that the changes in mitochondrial flux occurred earlier than the changes in resident AMs amount in AP-ALI. The data in the present study suggested that mitochondrial levels in AMs can be used as an index for ALI.

Similar to findings from confocal cell experiments, studies have shown that extensive mitochondrial damage leads to the removal of mitochondria through mitophagy, directing them to lysosomes 31. The results of the present study indicate that the levels of mitochondria in AMs can serve not only as an index for ALI but also as a significant target for its therapy. Supporting this perspective, research has demonstrated that enhancing mitochondrial function in AMs can alleviate ALI 32. Furthermore, the transfer of mitochondrial components via exosomes has been found to improve the mitochondrial integrity and oxidative phosphorylation in macrophages, thereby promoting metabolic and immune homeostasis in alveolar macrophages and reducing lung inflammatory pathology 33.

Another important finding is that, based on the pathological results of the lungs, EMO could only reduce the incidence of foci in a random field of view to a certain extent, rather than eliminate the foci of lung tissue. Our previous studies demonstrated that EMO impacts the progression of AP-ALI through its anti-inflammatory characteristics 18. In the present study, the mechanism through which EMO exerts its protective effects on mitochondrial homeostasis in AMs following AP-ALI was investigated. For that purpose, in vitro macrophage cell models treated with LPS/ATP were constructed. The results showed that EMO pretreatment significantly decreased the accumulation of uncleared damaged mitochondria and enhanced mitophagy in macrophages. This can have the following benefits. First, the greater the number of healthy mitochondria the better for the energy requests of activated immune cells. This would support the switch of cell metabolism from a resting state to a high metabolic state and maintain energy homeostasis. Then, maintaining normal permeability of the mitochondrial membrane by EMO supported the characteristics of anti-inflammation in macrophages. In addition, EMO reduced the accumulation of ROS and mtDNA in the cytoplasm (mtDAMPs) and further inhibited NLRP3 inflammasome formation (Figure S2 and 3). A change in mitochondrial flux was also observed and the relative number of AMs in AP-ALI was evaluated. Another study also reported that EMO could activate Parkin-mediated mitophagy and delay heart aging 34. Collectively, the present data elucidated the therapeutic effects of EMO on alleviating AP-ALI function through the promotion of mitophagy in AMs, which provided a novel therapeutic target and expanded out knowledge of EMO's beneficial effects in inflammatory diseases. Moreover, the study also led us to further understand the relationship between mitophagy and mitochondrial membrane potential. On one hand, as expounded in this article, the change of mitochondrial membrane potential is one of the important signs of mitochondrial damage. On the other hand, the changes in mitochondrial membrane potential are closely related to mitophagy. In PINK1-Parkin-mediated mitophagy, Parkin is recruited to depolarize mitochondria to promote autophagic degradation of mitochondria 35. In PINK1-Parkin-independent mitophagy: Upon conditions like hypoxia or loss of mitochondrial membrane potential, USP19 promotes the de-ubiquitination of FUNDC1, which promotes mitochondrial fission, leading to mitophagy 36.

## Conclusions

The present study indicated that i) mitochondrial status determined the fate of resident AMs and is critical in AP-ALI in mice; ii) monitoring and proactive intervention on mitophagy could protect mitochondrial damage and sequentially improve AP; iii) EMO protective effects in AP-ALI manifest as enhancing mitophagy. The present results suggested that EMO could be used as a reliable anti-inflammatory and mitophagy enhancer during AP-ALI, which should help improve the treatment of inflammatory diseases in the clinic.

## Supplementary Material

Supplementary figures and tables.
