# Supplementary material for: Emodin: an alveolar macrophage protector in acute pancreatitis induced lung injury
Source: Int J Med Sci. 2025 Mar 31;22(9):2075–87. doi: 10.7150/ijms.105965 (PMC12035834; doi:10.7150/ijms.105965)
Supplement: Supplementary file 1 — Supplementary figures and tables. [file ijmsv22p2075s1.pdf]

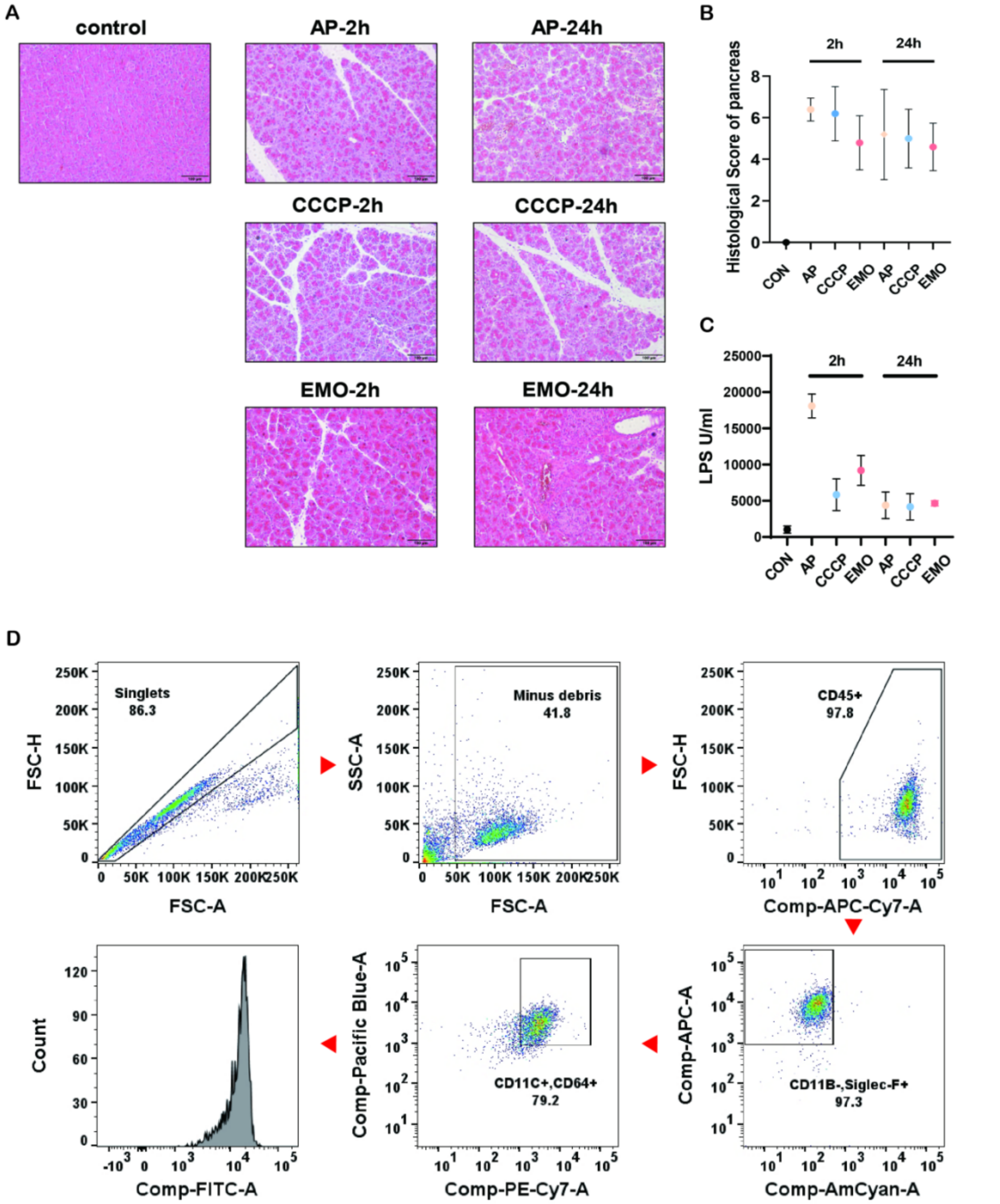

Figure S1 Pancreatic histopathological injury, score, lipase level and flow cytometry related to Figure 2B-D. (**A**,**B**) Representative histological images and quantification of pancreatic tissue injury, with or without CCCP and EMO administration (n=4 per group). (**C**) Lipase level analysis from A (n=4 per group). (**D**) Flow cytometry to identify AMs from cells collected in BALF.

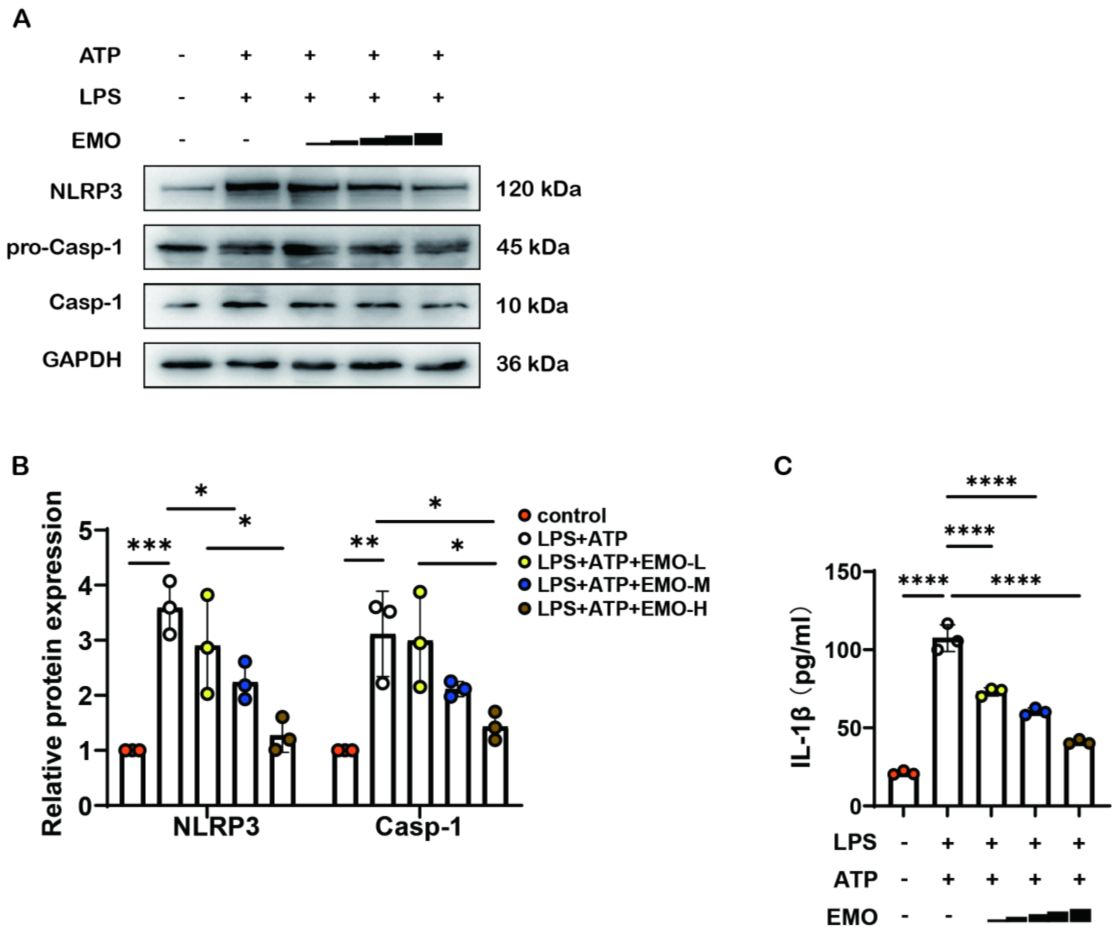

Figure S2 EMO reduces inflammation of AP-ALI in vitro. (**A**) Western blotting of NLRP3 and caspase-1 on protein lysates from MH-S pre-treated with various doses of EMO (2.5, 5 and 7.5 μM, 2 h), primed with LPS (500 ng/ml, 4 h) and followed by stimulation with ATP (2 mM, 1 h) (n=3 per condition). (**B**) Relative quantification of NLRP3 and caspase-1 in A. (**C**) Quantification of IL-1β in cultured supernatant pretreated with different doses of EMO (2.5, 5, 7.5 μM, 2 h) by ELISA assay. *P<0.05, **P<0.01, ***P<0.001 and ****P<0.0001.

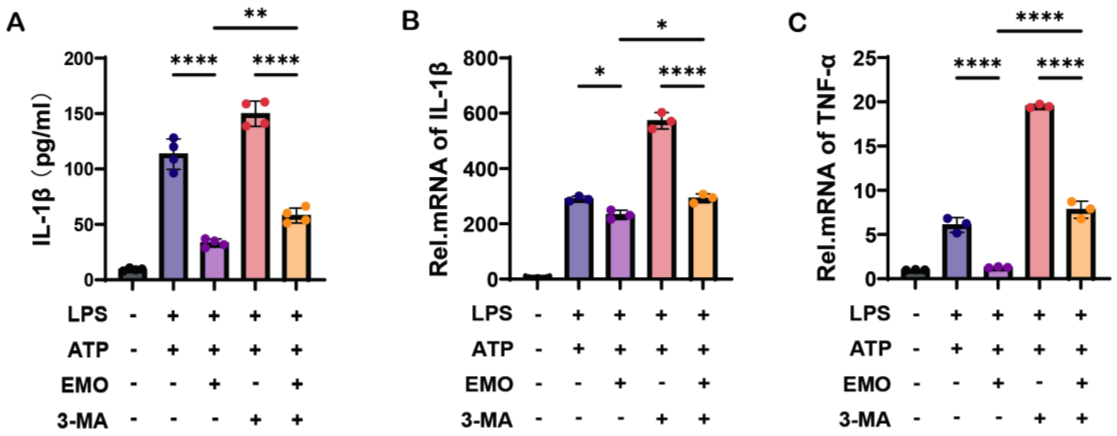

Figure S3 Selective inhibition of mitophagy affects EMO's impact on inflammatory cytokine secretion in vitro. (**A**) Quantification of IL-1β in MH-S culture supernatant with or without 3-MA treatment by ELISA. (**B**,**C**) qPCR analysis of IL-1β, TNF-α expression in MH-S (same treatement as in Figure 5D). *P<0.05, **P<0.01, ***P<0.001 and ****P<0.0001.

Table S1. Information on antibodies and experimental models.

Reagent or Resource	Source	Identifier
**Antibodies**		
APC-Cy7 Anti-Mouse CD45 (clone: 30-F11)	BD Bioscience	Cat# 561037; RRID: AB_396774
Fixable Viability Stain 700	BD Bioscience	Cat# 564997; RRID: AB_2869637
PE/Cyanine7 anti-mouse CD64 (clone: X54-5/7.1)	BioLegend	Cat# 139313; RRID: AB_2563903
BV510 Anti-Mouse CD11b (clone: M1/70)	BD Bioscience	Cat# 562950; RRID: AB_2737913
BV421 Hamster Anti-Mouse CD11c (clone: HL3)	BD Bioscience	Cat# 562782; RRID: AB_2737789
PE Anti-Mouse CD24 (clone: M1/69)	BD Bioscience	Cat# 553262; RRID: AB_394741
BB700 Anti-Mouse I-A/I-E (clone: 2G9)	BD Bioscience	Cat# 746086; RRID: AB_2743461
AF647 Anti-Mouse Siglec-F (clone: E50-2440)	BD Bioscience	Cat# 562680; RRID: AB_2687570
NLRP3 (D4D8T) Rabbit mAb	Cell signalling Technology	Cat# 15101S; RRID: AB_2722591
LC3A/B (D3U4C) Rabbit mAb	Cell signalling Technology	Cat# 12741; RRID: AB_2617131
GAPDH (D16H11) Rabbit mAb	Cell signalling Technology	Cat# 5174; RRID: AB_10622025
Rabbit Anti-GSDMD antibody	Abcam	Cat# ab209845; RRID: AB_2783550
Caspase 1/p20/p10 antibody	Proteintech	Cat# 22915-1-AP; RRID: AB_2876874
Goat Anti-Rabbit IgG H&L (Alexa Fluor® 488)	Abcam	Cat# ab150077; RRID: AB_2630356
Goat Anti-Mouse IgG H&L (Alexa Fluor 647)	Abcam	Cat# ab150115; RRID: AB_2687948
Anti-TOMM20 antibody Mouse IgG1	Abcam	Cat# ab283317
Anti-DRP1 antibody	Abcam	Cat# ab184247; RRID: AB_2895215
**Experimental Models: Cell Lines**		
Mouse: MH-S	Procell	Cat# CL-0597; RRID: CVCL_3855
Mouse: RAW264.7	Procell	Cat# CL-0190; RRID: CVCL_0493
**Experimental Models: Organisms**		
Mouse: C57BL/6J	ChangSheng Biotech	RRID: IMSR_JAX:000664

Table S2. Information on chemicals, peptides, and Recombinant Proteins.

Reagent or Resource	Source	Identifier
**Chemicals, Peptides, and Recombinant Proteins**		
Formalin	Absin	Cat# abs9179
ethylenediaminetetraacetic acid (EDTA)	MedChemExpress	Cat# HY-Y0682
BSA	BioFroxx	Cat# 4240
FBS	Newzerum	Cat# CE500
AG RNAex Pro RNA	Accurate Biology	Cat# AG21101
LPS (Escherichia coli O55:B5)	Sigma	Cat# L2880
MitoSOX Red	MedChemExpress	Cat# HY-D1055
MitoTracke Green FM	Invitrogen	Cat# M7514
Purified Rat Anti-Mouse CD16/CD32	BD Bioscience	Cat# 553141; RRID: AB_394656
Negative Control Compensation Particles Set	BD Bioscience	Cat# 552845; RRID: AB_10058522
Stain Buffer (FBS)	BD Bioscience	Cat# 554656
Lysing Buffer	BD Bioscience	Cat# 555899
Carbonyl cyanide 3-chlorophenylhydrazone (CCCP)	MedChemExpress	Cat# HY-100941
3-Methyladenine (3-MA)	MedChemExpress	Cat# HY-19312
RPMI 1640 (ATCC modification)	Gibco	Cat# A1049101
β-Mercaptoethanol Solution	Macklin	Cat# M917637
DMSO	Absin	Cat# abs9187
DAPI Staining Solution	Absin	Cat# abs47047616
ATP	MedChemExpress	Cat# HY-B2176
Mito-Tracker Deep Red 633	Beyotime	Cat# C1034
PMSF	Beyotime	Cat# ST506
RIPA Lysis Buffer	Beyotime	Cat# P0013C
Hoechst 33342	Beyotime	Cat# C1027
Propidium Iodide	Beyotime	Cat# ST511
Lyso-Tracker Red	Beyotime	Cat# C1046
Glutaraldehyde Fixed Solution	Absin	Cat# abs9277
Triton X-100	Elabscience	Cat# E-IR-R122
RNase A	Sangon Biotech	Cat# B600473
Ceruletide	MedChemExpress	Cat# HY-A0190
Emodin	Solarbid	Cat# E8390
MonAmp SYBR Green qPCR Mix	Monad	Cat# MQ10101S
MonScrip RTIII All-in-One Mix	Monad	Cat# MR05001S
Dulbecco’s modified Eagle’s medium (DMEM)	Gibco	Cat# 11995065

Table S3. Information on critical commercial assays, oligonucleotides, and software.

Reagent or Resource	Source	Identifier
**Critical Commercial Assays**		
Mouse Cholesterol Kit	Como Bio	Cat# CB10360
PKH26PCL fluorescent cell linker kit	Sigma	Cat# PKH26PCL
Mitochondrial membrane potential assay kit with JC-1	Beyotime	Cat# C2006
Cell Counting Kit-8 (CCK-8)	Apexbio	Cat# K1018
ONE-4-ALL Genomic DNA Mini-Preps Kit	BioBasic	Cat# B618503
ROS Assay Kit	Beyotime	Cat# S0033S
LDH Cytotoxicity Assay Kit	Beyotime	Cat# C0016
MS Mouse IL-1β ELISA Kit	Elabscience	Cat# E-MSEL-M0003
MS Mouse TNF-α ELISA Kit	Elabscience	Cat# E-MSEL-M0002
BCA Protein Assay Kit	Keygen Biotech	Cat# KGP902
**Oligonucleotides**		
Mouse： *IL1β* Forward GCAACTGTTCCTGAACTCAACT	PrimerBank	6680415a1
Mouse： *IL1β* Reverse ATCTTTTGGGGTCCGTCAACT	PrimerBank	6680415a1
Mouse： *D-loop* Forward AATCTACCATCCTCCGTGAAACC	PMID: 36522512	N/A
Mouse： *D-loop* Reverse TCAGTTTAGCTACCCCCAAGTTTAA	PMID: 36522512	N/A
Mouse： *Tert* Forward CTAGCTCATGTGTCAAGACCCTCTT	GenBank Accession	NM_009354
Mouse： *Tert* Reverse GCCAGCACGTTTCTCTCGTT	GenBank Accession	NM_009354
Mouse： *Actb* Forward GTGACGTTGACATCCGTAAAG	PrimerBank	145966868c1
Mouse： *Actb* Reverse GCCGGACTCATCGTACTCC	PrimerBank	145966868c1
Mouse： *Tnf* Forward CCTGTAGCCCACGTCGTAG	PrimerBank	133892368c3
Mouse： *Tnf* Reverse GGGAGTAGACAAGGTACAACCC	PrimerBank	133892368c3
**Software**		
FlowJo Software	TreeStar	https://www.flowjo.com/
GraphPad Prism v8 software	GraphPad	https://www.graphpad.com/
ImageJ Software	NIH	http://www.imagej.nih.gov/ij/
Case Viewer	3DHISTECH	https://www.3dhistech.com/
CellSens	Olympus	https://lifescience.evidentscientific.com.cn/zh/
Leica Application Suite	Leica	Advanced Fluorescence
BD FACSDiva	BD Bioscience	Version 9.0
